# Supplementary material for: A versatile functional interaction between electrically silent KV subunits and KV7 potassium channels
Source: Cell Mol Life Sci. 2024 Jul 14;81(1):301. doi: 10.1007/s00018-024-05312-1 (PMC11335225; doi:10.1007/s00018-024-05312-1)
Supplement: Supplementary file 1 — Supplementary file1 (PDF 4826 KB) [file 18_2024_5312_MOESM1_ESM.pdf]

Supplementary Figures

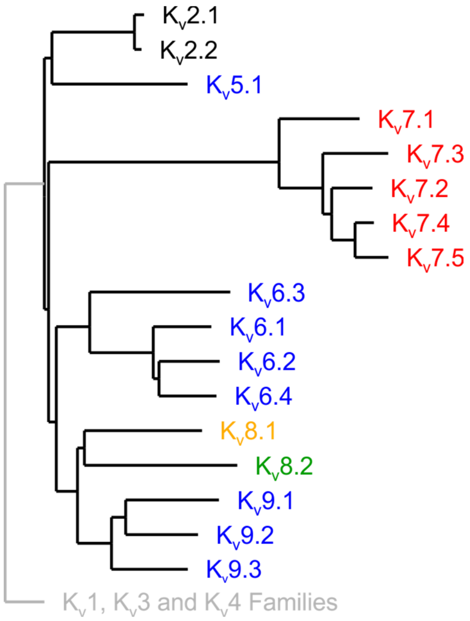

Supplementary Figure 1: **Phylogenetic tree of Kv channels.** Alignment was performed on the core region (S1-S7) of the human Kv orthologs as listed by IUPHAR using clustalW2. Phlogenetic trees were caluclated using R and the ape package. Distance matrices were calculated using the maximum likehood method and trees were calculated using the BIONJ algorithm. Kv channel family members are color coded as red (Kv7) black (Kv2) and blue (KvS). Silent Kv8 are highlighted in orange (Kv8.1) and green (Kv8.2), respectively.

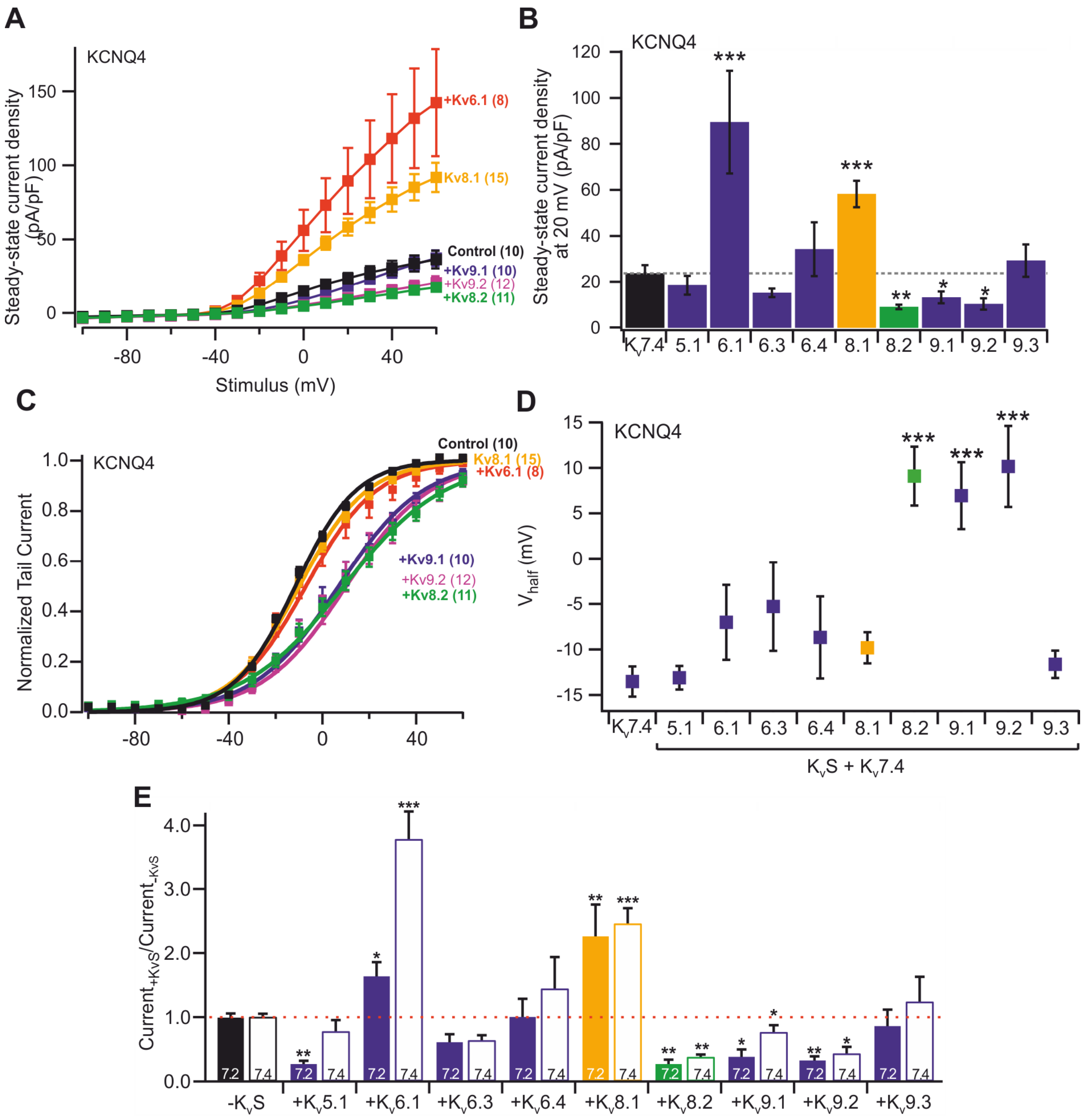

Supplementary Figure 2 (expanding on Figure 1): Electrophysiological properties of CHO cells expressing Kv7.4 channels are altered by co-expression of KvS. **(A)** Voltage-dependent current densities in cells expressing Kv7.4 alone (black), and together with different KvS. **(B)** Summary statistics for steady-state current densities at +20mV obtained in recordings similar to those shown in (A). **(C)** Normalized tail currents and half-maximal activation voltage (D) as deduced from Boltzmann fits (solid lines in C) for the same cells as shown in A and B. **(E)** Steady-state current densities at +20mV for Kv7.2 (solid bars) or Kv7.4 (empty bars) co-transfected with the indicated KvS. Data are shown as relative current densities normalized to the mean of current densities observed when Kv7.2 or Kv7.4 when transfected alone ("-KvS").

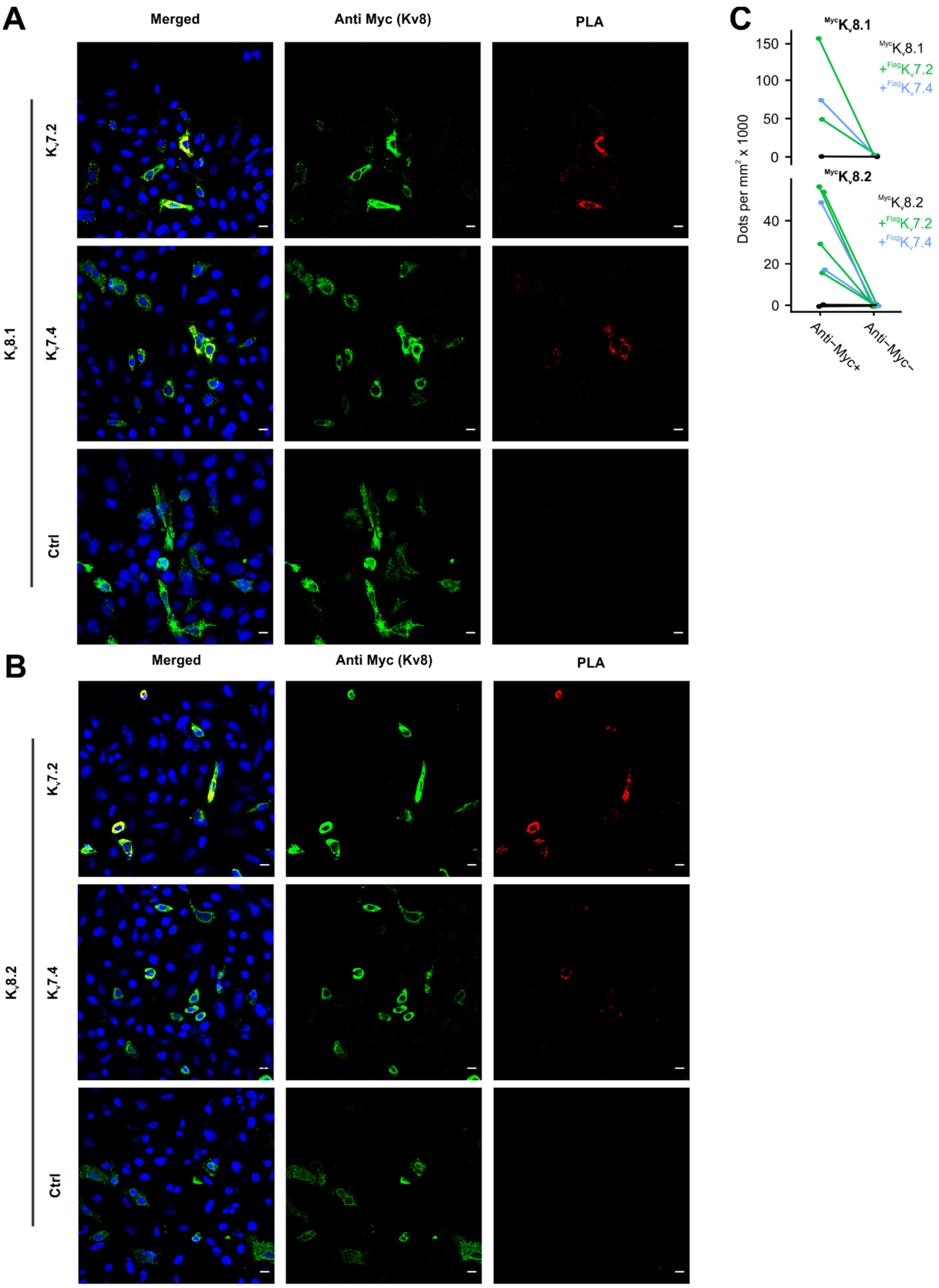

Supplementary Figure 3: Kv7 and KvS exist in close proximity in intact cells. (A+B) Combined immunocytochemistry and proximity ligation essay (PLA) of cells co-transfected with myc-tagged Kv8.1 **(A)** or myc-tagged Kv8.2 **(B)** and either flag-tagged Kv7.2 (first row) or Kv7.4 (second row) or alone (third row). Green channel: anti-myc (Kv8.1 and Kv8.2, resp.), red channel: PLA, blue channel: DAPI. In PLA, physical proximity of two proteins is detected by ligating and amplifying oligonucleotide probes that are attached to the secondary antibodies, followed by binding of fluorescent probes. Ligation can only occur if probes are in close proximity (several nanometers). In

these experiments, PLA signal can only be observed in cells immunopositive for Myc (green, demonstrating successful expression of Kv8) when also a Kv7 subunit was co-expressed. **(C)** Summary statistics for experiments shown in A and B. Shown is the number of PLA dots per mm² inside cells immunopositive for myc vs. the number of PLA dots per mm² outside cells immunopositive for myc in the same field of view. Data points from cells transfected with KvS alone are drawn in black, from those transfected together with flag-tagged Kv7.2 or Kv7.4 in light green and light blue, respectively. Scale bar: 10 µm.

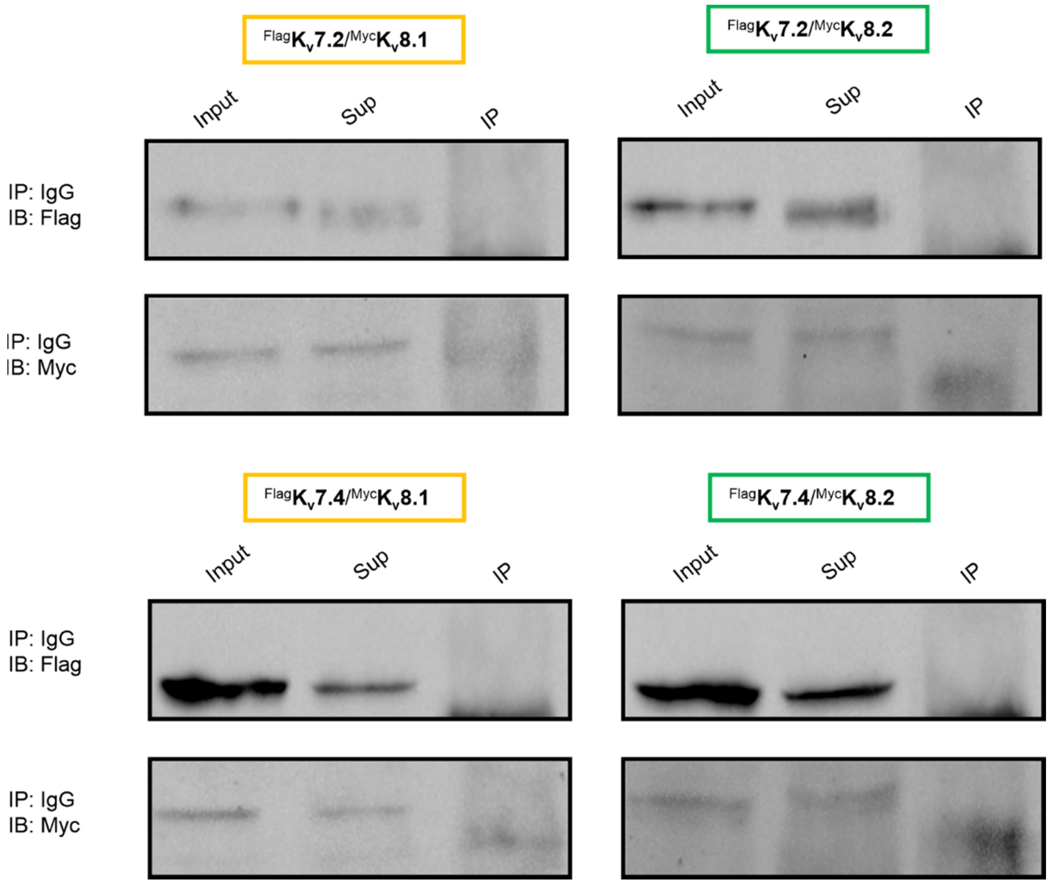

Supplementary Figure 4: Co-immunoprecipitation of flag tagged Kv7 and myc tagged KvS subunits using unrelated IgG antibodies. HEK cells were co transfected with flag tagged Kv7.2 **(A)** and Kv7.4 **(B)** and myc tagged Kvs subunits and the lysates were precipitated with Dynabeads coated with unrelated IgG antibodies and blotted with anti-flag (Kv7.2) and anti-myc (KvS) antibodies respectively. Note the absence of myc tagged KvS subunits in the IP fraction, although epitope tagged Kv7 and KvS subunits were expressed and detected in both input and supernatant (Sup) fractions. Abbreviations used: IP (Immunoprecipitation), IB (immunoblotting), Sup (supernatant).

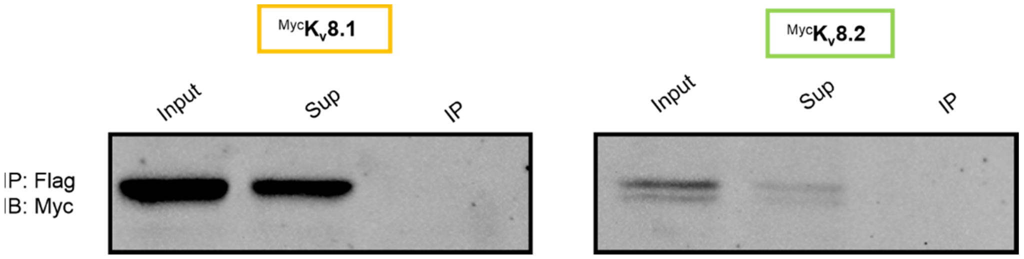

Supplementary Figure 5: Co-immunoprecipitation of myc tagged Kvs subunits alone with flag antibody coated Dynabeads). HEK cells were transfected with myc tagged Kvs subunits alone (absence of flag tagged Kv7

subunits) and the lysates were precipitated with flag beads and blotted with anti-myc (K_vs) antibodies. Abbreviations used: IP (Immunoprecipitation), IB (immunoblotting), Sup (supernatant).

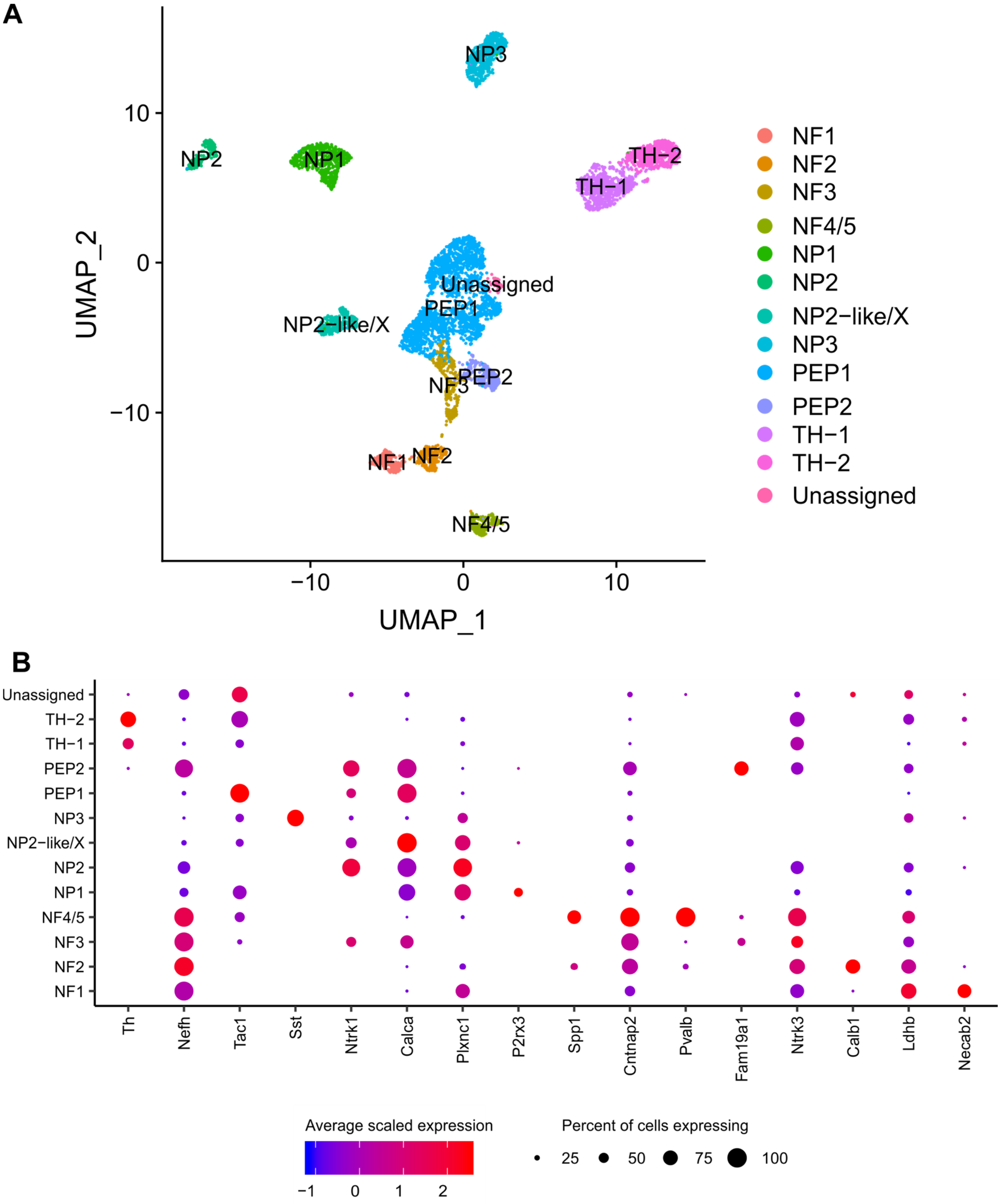

Supplementary Figure 6 (expanding on Figure 6): Unsupervised clustering of a dorsal root ganglia single-cell RNA sequencing dataset. (A) Uniform Manifold Approximation and Projection (UMAP) visualization of the transcriptional heterogeneity of 6585 dorsal root ganglia cells. Colors represent cluster assignments, determined using a shared nearest neighbor (KNN) modularity optimization-based clustering approach. Cluster were assigned names according on their expression of marker genes as shown in B. **(B)** Expression dot plot of published marker

genes for DRG cell types. Colour codes for the average scaled expression of the respective gene in each cluster and dot size codes for the percentage of cells in which transcripts of that gene where detected in.

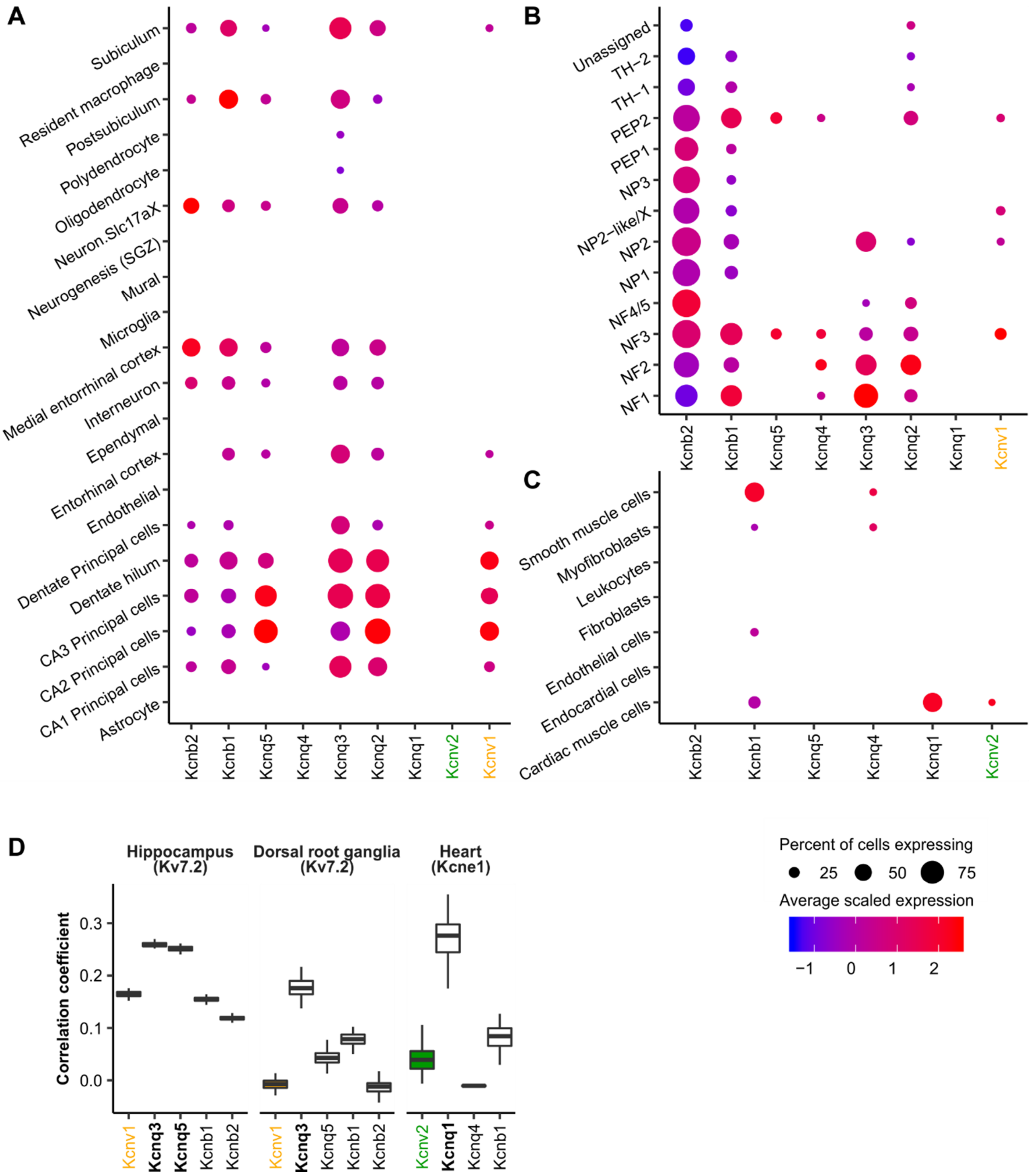

Supplementary Figure 7 (expanding on Figure 6): Co-expression of K_V genes in Hippocampus, Dorsal root ganglia and Heart. (A-C) Expression dot plots of K_VS, K_V7 and K_V2 genes, for comparison, in Hippocampus (A), Dorsal root ganglia (B) and Heart cells (C), respectively. Expression dot plot of published marker genes for DRG cell types. Colour codes for the average scaled expression of the respective gene in each cluster and dot size codes for the percentage of cells in which transcripts of that gene where detected in. **(D)** Correlation coefficients for the single-cell expression of K_V7.2 in Hippocampus and Dorsal root ganglia and Kcne1 in Heart cells. These are well established to assemble with K_V7.3 and K_V7.1 channels, respectively. These results can be considered as a positive control for the assertion that for K_V-channels protein interaction can be inferred from transcript count correlation. Shown are Pearson's correlation coefficients for log2+1-transformed transcript counts from individual cells as observed in three publicly available singe-cell RNA sequencing datasets. Included into the analysis were the same cell types as in Figure 4 C.

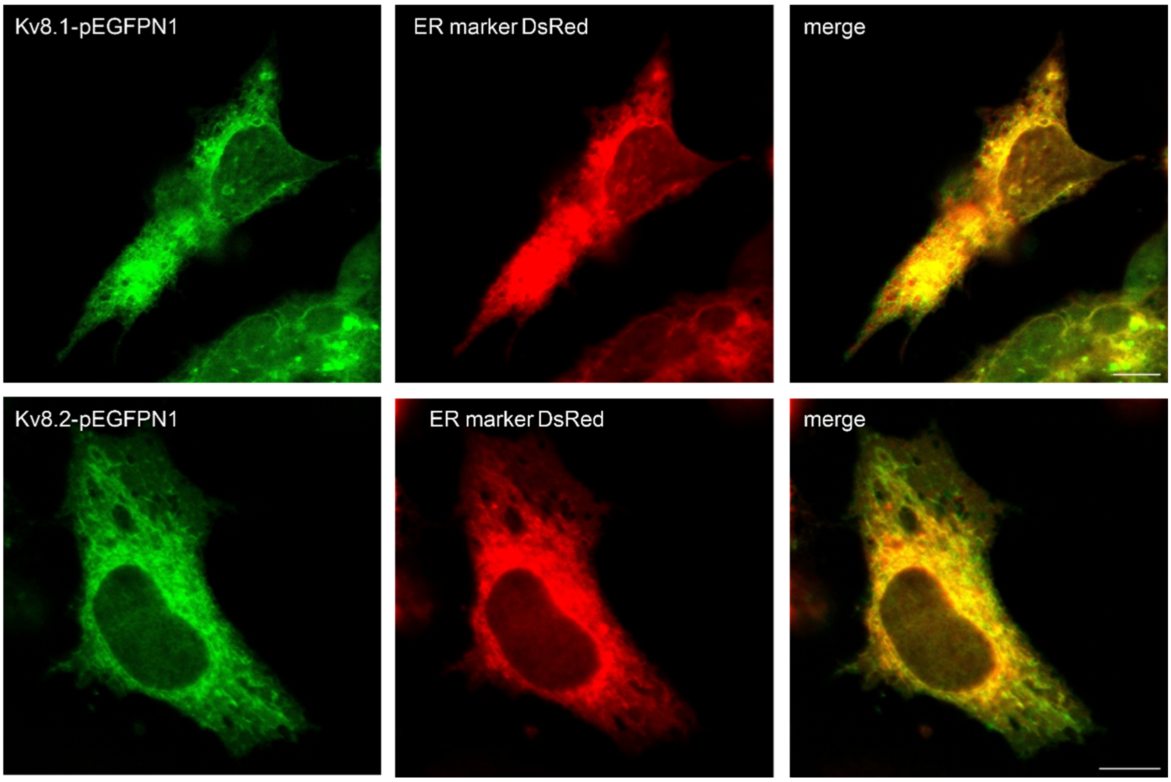

Supplementary Figure 8: GFP tagged KvS subunits colocalise with an ER marker. HeLa cells were co-transfected with GFP-tagged KvS and DsRed tagged ER marker. Note that the GFP-KvS reticular pattern (left) overlaps with DsRed2-ER (centre). This can be seen in the merged image (right). All images were taken 48 h post-transfection. Similar results were obtained in n = 3 transfections. Scale bars: 10 µm.
