# Supplementary material for: A versatile functional interaction between electrically silent KV subunits and KV7 potassium channels
Source: Cell Mol Life Sci. 2024 Jul 14;81(1):301. doi: 10.1007/s00018-024-05312-1 (PMC11335225; doi:10.1007/s00018-024-05312-1)
Supplement: Supplementary file 2 — Supplementary file2 (PDF 936 KB) [file 18_2024_5312_MOESM2_ESM.pdf]

Supplement material 1: Single cell RNA sequencing

Figure S1: Distribution of Marker genes for cell type assignment of individual clusters and reference

Table S1: Per-cell read and transcript counts in the three datasets analysed:

	DRG	HEART	HIPPOCAMPUS
**READS**	4260.00 [2755.00 - 7130.00]	832795.00 [514635.00 - 1180777.00]	3093.00 [2142.00 - 4867.00]
**TRANSCRIPTS**	1963.00 [1480.00 - 2667.00]	2611.00 [1943.00 - 3317.00]	1761.00 [1298.00 - 2447.00]

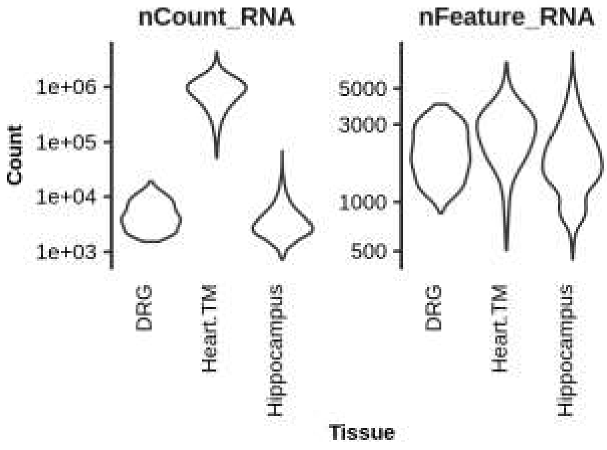

Figure S1: Violin plot corresponding to Table S1.

Per-cell read and transcript counts for the three datasets analysed. Note the difference that the Heart dataset was based on RNA-sequencing from cells FAC-sorted into microwell plates, resulting in a higher read depth as compared to the droplet-sequencing technique that was used for the other datasets.

Table S2: Read counts for Kv and KvS channels of interest and the percentage of cells with at least one read in the three datasets analysed:

	DRG		HEART		HIPPOCAMPUS	
	N	%	N	%	N	%
**KCNB1**	2035	30.9	346	7.9	13090	24.8
**KCNB2**	5050	76.7	5	0.1	9710	18.4
**KCNQ1**	10	0.2	161	3.7	72	0.1
**KCNQ2**	909	13.8	NA	0	16743	31.7
**KCNQ3**	655	9.9	NA	0	24231	45.9
**KCNQ4**	207	3.1	101	2.3	411	0.8

**KCNQ5**	403	6.1	14	0.3	7861	14.9
**KCNV1**	446	6.8	0	0	8395	15.9
**KCNV2**	0	0	39	0.9	3	0

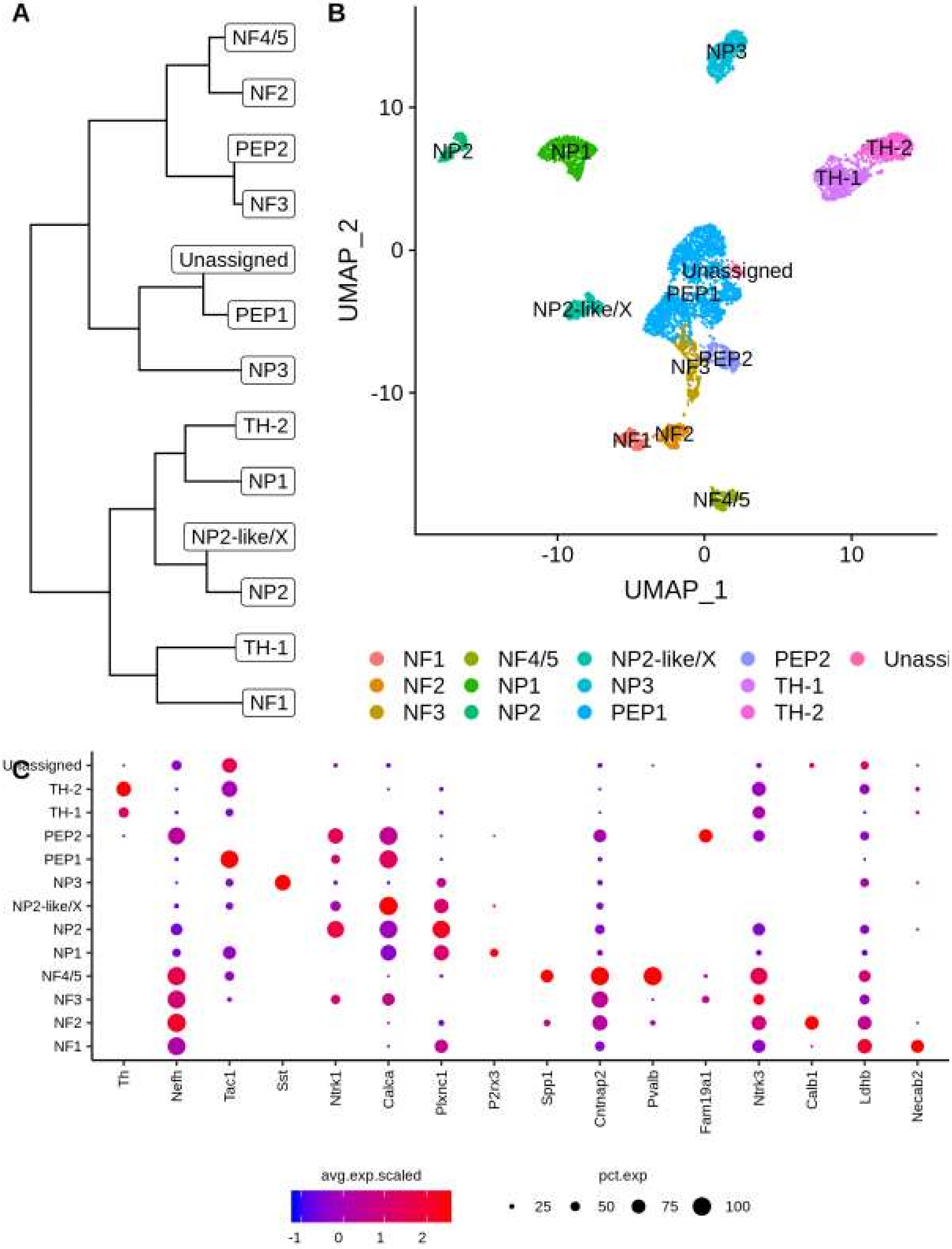

Figure S3: Clustering of the DRG dataset.

As cluster assignments for the cells contained in the DRG dataset from Finno et al were not available clustering was re-performed using the first eighteen principal components obtained after regressing

out technical variance. The cluster “PEP1” resulted from merging three sub-clusters that all seemed to represent PEP1 cells. A) Cluster tree as obtained using the K-nearest neighbour method and the Louvain algorithm. B) Obtained clusters (colours) match the grouping observed by Uniform Manifold Approximation and Projection (UMAP). Clusters were assigned a corresponding cell type analysing the overlap between the top 10 differentially expressed genes identified in each cluster and the differentially genes identified and kindly provided by Carrie Finno. C) As an independent validation of the obtained cell type assignments expression of a set of marker genes as reported by Usoskin et al (PMID: 25420068) was mapped.

Table S3 A: Pearson correlation coefficients for the normalized read counts of Kv8 with Kv7 and Kv2 channels

TISSUE	KCNQ1	KCNQ2	KCNQ3	KCNQ4	KCNQ5	KCNB1	KCNB2
**HIPPOCAMPUS (KV8.1)**	NA	0.16 [0.16 - 0.17]	0.18 [0.18 - 0.19]	NA	0.16 [0.16 - 0.16]	0.07 [0.06 - 0.07]	0.05 [0.04 - 0.05]
**DORSAL ROOT GANGLIA (KV8.1)**	NA	-0.01 [-0.02 - -0.00]	-0.01 [-0.02 - 0.00]	NA	0.04 [0.03 - 0.05]	0.06 [0.05 - 0.07]	0.06 [0.05 - 0.07]
**HEART (KV8.2)**	0.11 [0.09 - 0.14]	NA	NA	-0.01 [-0.01 - -0.00]	NA	0.01 [-0.00 - 0.02]	NA

Table S3 B: Percentile distributions corresponding to table S3 A

TISSUE	KCNQ1	KCNQ2	KCNQ3	KCNQ4	KCNQ5	KCNB1	KCNB2
**HIPPOCAMPUS (KV8.1)**	NA	0.99 [0.98 - 0.99]	1.00 [0.99 - 1.00]	NA	0.98 [0.98 - 0.99]	0.69 [0.68 - 0.71]	0.58 [0.56 - 0.60]
**DORSAL ROOT GANGLIA (KV8.1)**	NA	0.31 [0.23 - 0.38]	0.28 [0.20 - 0.41]	NA	0.83 [0.73 - 0.88]	0.94 [0.90 - 0.97]	0.95 [0.91 - 0.97]
**HEART (KV8.2)**	0.99 [0.98 - 1.00]	NA	NA	0.22 [0.20 - 0.26]	NA	0.36 [0.26 - 0.46]	NA

Table S4 A: Pearson correlation coefficients for the normalized read counts of reported heteromerization partners of Kv7 channels in the different tissues to validate the biological plausibility of the approach

TISSUE	KCNV1	KCNV2	KCNQ1	KCNQ3	KCNQ4	KCNQ5	KCNB1	KCNB2
**HIPPOCAMPUS (KV7.2)**	0.16 [0.16 - 0.17]	NA	NA	0.26 [0.26 - 0.26]	NA	0.25 [0.25 - 0.25]	0.15 [0.15 - 0.16]	0.12 [0.12 - 0.12]
**DORSAL ROOT GANGLIA (KV7.2)**	-0.01 [-0.01 - -0.00]	NA	NA	0.18 [0.16 - 0.19]	NA	0.04 [0.03 - 0.05]	0.08 [0.07 - 0.09]	-0.01 [-0.02 - -0.01]
**HEART (KCNE1)**	NA	0.04 [0.02 - 0.06]	0.28 [0.24 - 0.30]	NA	-0.01 [-0.01 - -0.01]	NA	0.08 [0.07 - 0.10]	N

Table S4 B: Percentile distributions corresponding to table S4 A

TISSUE	KCNV1	KCNV2	KCNQ1	KCNQ3	KCNQ4	KCNQ5	KCNB1	KCNB2
**HIPPOCAMPUS (KV7.2)**	0.84 [0.83 - 0.85]	NA	NA	0.97 [0.97 - 0.98]	NA	0.97 [0.97 - 0.97]	0.82 [0.81 - 0.83]	0.71 [0.70 - 0.72]
**DORSAL ROOT GANGLIA (KV7.2)**	0.30 [0.26 - 0.35]	NA	NA	1.00 [1.00 - 1.00]	NA	0.64 [0.59 - 0.71]	0.84 [0.80 - 0.87]	0.27 [0.23 - 0.31]
**HEART (KCNE1)**	NA	0.54 [0.45 - 0.65]	1.00 [1.00 - 1.00]	NA	0.27 [0.27 - 0.28]	NA	0.77 [0.68 - 0.82]	NA

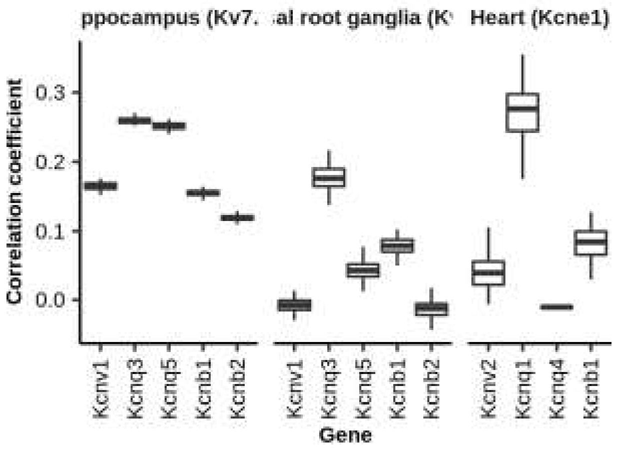

Figure S4 related to Table S4 A.

Correlation coefficients of the scaled read counts of Kcnq2 (for Hippocampus and DRG) and Kcne1 (for heart) with those for all Kcnq, Kcnb and Kcnv genes.
