# Supplementary material for: A versatile functional interaction between electrically silent KV subunits and KV7 potassium channels
Source: Cell Mol Life Sci. 2024 Jul 14;81(1):301. doi: 10.1007/s00018-024-05312-1 (PMC11335225; doi:10.1007/s00018-024-05312-1)
Supplement: Supplementary file 3 — Supplementary file3 (DOCX 19 KB) [file 18_2024_5312_MOESM3_ESM.docx]

Supplementary Table 1

|  | **Gene name** | **Accession nr.** | **Construct** |
| --- | --- | --- | --- |
| **Electrophysiology** | KCNQ2 | NM_172107.4 | - Kv7.2/pBK/CMV |
|  | KCNQ4 | NM_172163.3 | - Kv7.4/pBK/CMV |
|  | KCNV1 | NM_026200.3 | - Kv8.1/pBK/CMV |
|  | KCNV2 | NM_133497.4 | - Kv8.2/pBK/CMV |
|  | KCNF1 | NM_002236.4 | - Kv5.1/ pBK/CMV |
|  | KCNG1 | NM_002237.3 | - Kv6.1/pCDNA3.1 |
|  | KCNG3 | NM_133329.5 | - Kv6.3/pCDNA3.1 |
|  | KCNG4 | NM_172347.2 | - Kv6.4/pCDNA3.1 |
|  | KCNS1 | NM_008435.2 | - Kv9.1/pCDNA3.1 |
|  | KCNS2 | NM_001271704.1 | - Kv9.2/pCDNA3.1 |
|  | KCNS3 | NM_001282428.2 | - Kv9.3/pCDNA3.1 |
| **Surface quantification assay** | KCNQ2 | NM_172107.4 | - Kv7.2^HA^-pSGEM |
|  | KCNQ4 | NM_172163.3 | - Kv7.4^HA^-pSGEM |
|  | KCNV1 | NM_026200.3 | - Kv8.1-pSGEM |
|  | KCNV2 | NM_133497.4 | - Kv8.2-pSGEM |
| **Co-immunoprecipitation assay** | KCNQ2 | NM_172107.4 | - Kv7.2-flag-pCDNA3.1 |
|  | KCNQ4 | NM_172163.3 | - Kv7.4-flag-pCDNA3.1 |
|  | KCNV1 | NM_026200.3 | - mycKv8.1--pCDNA3.1 |
|  | KCNV2 | NM_133497.4 | - mycKv8.2--pCDNA3.1 |
| **BioID proximity assay** | KCNQ2 | NM_172107.4 | - Kv7.2-flag-pCDNA3.1 |
|  | KCNV1 | NM_026200.3 | - Kv8.1-BioID2-HA-pCDNA3.1 |
|  | KCNV2 | NM_133497.4 | - Kv8.2-BioID2-HA-pCDNA3.1 |
|  |  | WP_010880335.1 | - BioID2-HA-pCDNA3.1 |
| **PLA assay** | KCNV1 | NM_133497.4 | - mycKv8.1--pCDNA3.1 |
|  | KCNV2 | NM_026200.3 | - mycKv8.2--pCDNA3.1 |
| **Live cell imaging** | KCNV1 | NM_133497.4 | - Kv8.1-pEGFPN1 |
|  | KCNV2 | NM_026200.3 | - Kv8.2-pEGFPN1 |
